# Case report: Fatal traumatic coronary artery dissection—an overlooked complication of chest fracture

**DOI:** 10.3389/fcvm.2023.1226129

**Published:** 2023-09-05

**Authors:** Tao Jiang, Cheng Qian, Gang Wei, Ling Cheng, Wenwu Zheng, Gong Chen

**Affiliations:** ^1^ Depatment of Cardiology, The Affiliated Hospital of Southwest Medical University, Luzhou, China; ^2^ Depatment of Infection Managrment, The Affiliated Hospital of Southwest Medical University, Luzhou, China

**Keywords:** coronary artery dissection, trauma, chest, fracture, OCT

## Abstract

Coronary artery dissection caused by trauma is a rare occurrence that can be life-threatening. Accordingly, its rapid identification and treatment are essential to improve patient outcomes. Here, we present a case of a patient who suffered multiple rib and femur fractures after falling from a height of eighteen meters and subsequently experienced persistent chest pain. After the initial diagnostic workup, the medical team diagnosed the patient's chest pain as rib fractures and failed to consider the potential of a cardiac injury as the underlying cause. No emphasis was placed on monitoring changes in myocardial enzymes and ECG, which could have indicated coronary artery dissection. The dissection was confirmed and treated with a stent only after the subsequent coronary angiography (CAG) and optical coherence tomography (OCT) examinations, gradually relieving the patient's chest pain. In this case report, we discuss the management of fractures complicated by traumatic coronary artery dissection and highlight the benefits of OCT in diagnosing and treating this condition. The case also emphasizes the importance of considering coronary artery injury in patients with chest pain due to trauma.

## Introduction

Coronary artery dissection (CAD) is a cardiovascular condition that can cause acute myocardial ischemia, leading to abnormal cardiac enzyme levels and ST-segment changes similar to those typically observed in acute coronary syndrome (1). Coronary artery dissection can be divided into spontaneous coronary artery dissection (SCAD) and secondary coronary artery dissection. Secondary coronary artery dissection including invasive coronary procedures and trauma, is more common than SCAD.

During Coronary angiography (CAG) and percutaneous coronary intervention (PCI), canting of the guiding catheter, mismatched balloon type and dilation pressure, and repeated entry and exit of the catheter guide wires into and out of the vessel can all lead to coronary artery dissection (2). Coronary artery dissection caused by trauma has rarely been reported in the literature, given its low incidence and insidious nature (3). The paper addresses the conflicting matters related to the precedence of coronary artery dissection surgery compared to other surgical procedures. Additionally, it examines the use of anti-coagulation and coagulation treatment, which lack a clear recommendation in the current guidelines and literature. A case report is presented to explore management options for fractures associated with traumatic coronary artery dissection, and the advantages of optical coherence tomography (OCT) in treating dissections are also highlighted.

## Case presentation

**Figure 1:**
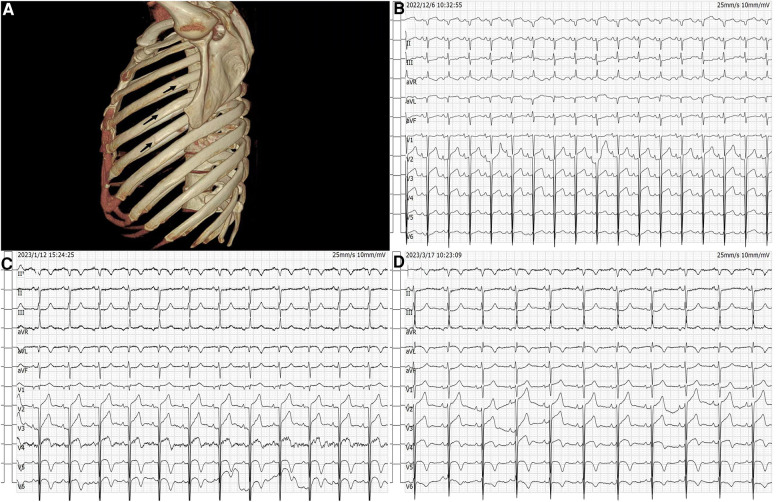
The fracture site and dynamic changes of electrocardiogram. (**A**) Left 4th–6th rib fracture indicated by the arrow. (**B**) Admission electrocardiogram. (**C**) Post PCI electrocardiogram. (**D**) Discharge electrocardiogram.

A 48-year-old male patient was hospitalized with fractures involving multiple body regions resulting from a fall injury that occurred more than ten days prior. The patient fell from a height of eighteen meters on a construction site. He was a non-smoker without a history of hypertension, diabetes and hyperlipidemia. Emergency CT examination revealed discontinuity of the left axillary segment of the 4th–6th rib (Figure 1A); discontinuity of the right anterior segment of the 6th–7th rib, axillary segment, and posterior segment of the 8th-9th rib; fracture of the left transverse process of L1-2 and L4-5; fracture of the pelvis; multiple fractures of the left upper femur; fracture of the left tibial plateau and left foot. Upon admission, an ECG suggested QS pattern and ST-segment elevation in leads V1–V4, I, and aVL (Figure 1B). Cardiac ultrasound showed no obvious abnormalities.

**Figure 2:**
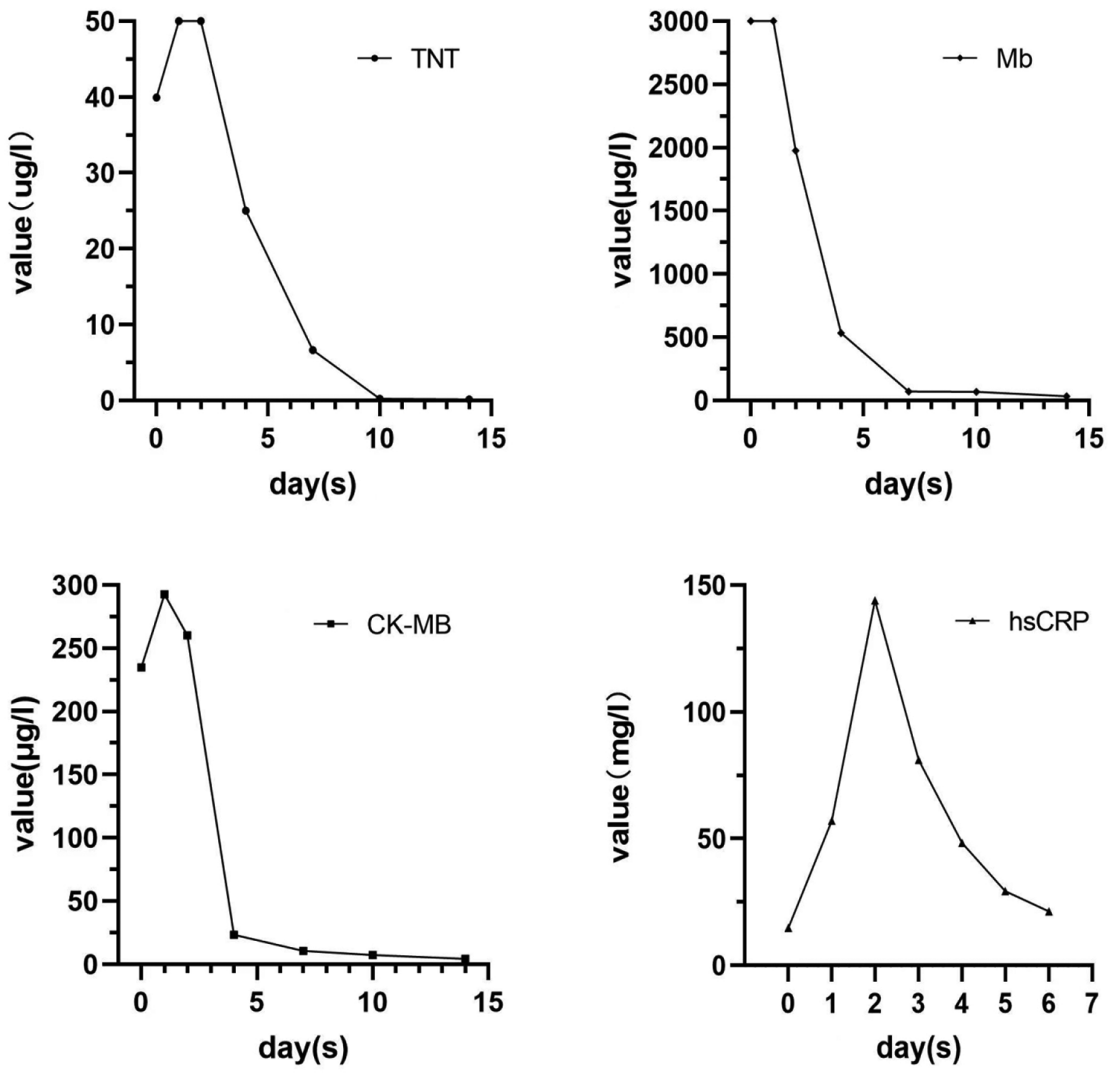
Dynamic changes in cardiac enzymes and hsCRP during hospitalization.

**Figure 3:**
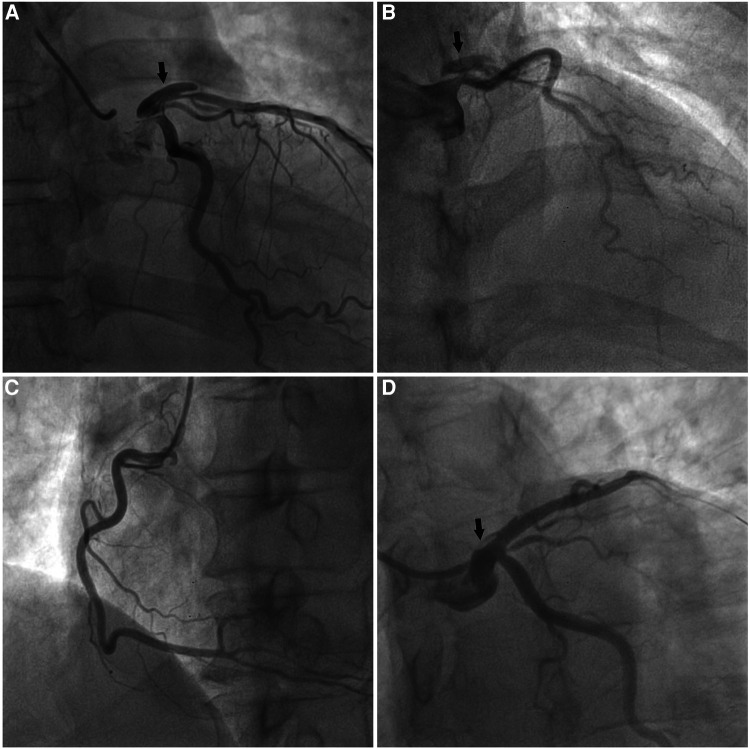
Coronary angiography of the patient. (**A,B**) The arrow showed a dissection in the left anterior descending artery, and no stenosis was seen in the left circumflex artery. (**C**) The right coronary artery was normal. (**D**) Coronary angiography was repeated after stenting, and no luminal stenosis was seen at the arrow, and the dissection was closed.

**Figure 4:**
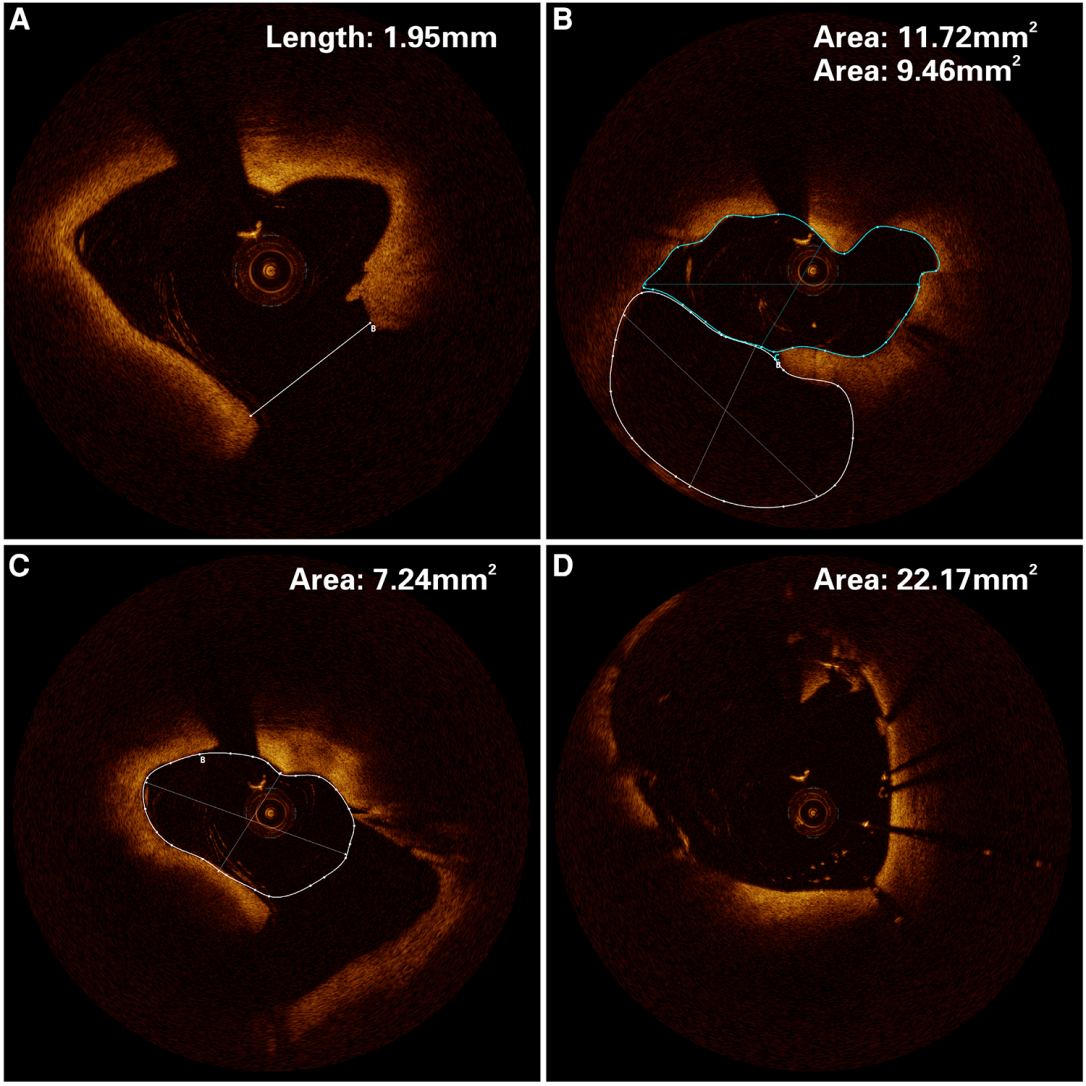
Optical coherence tomography of the patient. (**A**) An opening tear length of 1.95 mm in the left anterior descending artery. (**B**) The solid white line is the dissection hematoma, and the solid blue line is the true lumen area of the vessel, with an area of 11.72 mm^2^ and 9.46 mm^2^ respectively. (**C**) The white solid line area is the minimum area of 7.99 mm^2^ for the real lumen. (**D**) The stent was well apposed to the wall, with an area of 22. 17 mm^2^.

Upon admission, the patient complained of pain in multiple body areas, including bilateral crushing pain in the chest lasting approximately 30 min. An Ibuprofen-Codeine Sustained Tablet was administered to relieve the pain during severe episodes. The patient had elevated levels of myocardial enzymes and hsCRP prior to admission, and the changes observed during subsequent repeat tests suggest a progressive development of myocardial infarction. As a result, the possibility of acute myocardial infarction was considered upon review of the case data (Figure 2). The results of coronary angiography performed on 2023-01-06 showed approximately 90% LM-LAD stenosis, TIMI grade 3 antegrade flow, and a large convex lumen with a clear, high-bright band visible in the vessel lumen, which suggested the presence of a dissection hematoma compressing the proximal segment lumen. The left circumflex artery (LCX) and right coronary artery (RCA) were normal (Figures 3A–C). OCT of the coronary artery showed LAD intimal tear flaps, false lumen ruptures, and intramural hematomas visible at the opening. However we didn't find plaques. The dimensions of the coronary artery dissection were as follows: the length of the opening tear was 1.95 mm, the length of the entire dissection was 14.5 mm, the maximum area of the hematoma was 11.72 mm^2^, the area of the true lumen was 9.46 mm^2^, and the minimum true lumen area at the site of the dissection was 7.24 mm^2^ (Figures 4A–C). A stent was implanted at the proximal end of the LAD and across the end of the LM. A balloon was used for hyperbaric dilation. A repeated angiography indicated that the stent was successfully deployed, and there was TIMI3 forward blood flow (Figure 3D). A repeat OCT showed good stent apposition against the wall, with no dissection or intracavitary hematoma (Figure 4D). Postoperatively, the patient was treated with rivaroxaban tablets 15 mg once a day for DVT. Indobufen 0.1 g twice daily was selected due to its reversible inhibition of COX-1, lower impact on prostacyclin, reduced gastrointestinal adverse effects, lower bleeding risk, and shorter half-life compared to aspirin (4). A postoperative electrocardiogram showed QS pattern and ST-segment elevation in leads V1–V6, I, and aVL, and T-wave inversion in leads V4–V6, I, and aVL (Figure 1C). The patient's chest pain improved after stenting. On 2023-02-03, the patient underwent fracture reduction, and indobufen was stopped 24 h before the surgery. Tirofiban was administered as a bridge therapy 4 h prior to the surgery. The patient had a smooth recovery. A repeat electrocardiogram indicated ST segment depression in leads V1–V6, I, and aVL and T-wave inversion flattening in leads V4–V6, I, and aVL (Figure 1D) (compared with admission). The cardiac ultrasound did not reveal any significant abnormalities in the intracardiac flow that could lead to the development of a ventricular aneurysm. Thromboelastography (TEG) showed no significant abnormalities. After discharge, the patient was given rivaroxaban tablets 15 mg Qd, aspirin 100 mg Qd, and rosuvastatin 20 mg Qn.

## Discussion

Traumatic coronary artery dissection is a serious condition not well documented in the medical literature regarding its risk factors, pathophysiological mechanisms, and mechanical composition despite being rare. Unlike spontaneous coronary artery dissection or dissections related to percutaneous coronary intervention, traumatic dissections are usually the result of blunt trauma to the chest wall, such as chest compression, violent chest impact, or a fall from a height (5). The occurrence of traumatic coronary artery dissection is closely linked to the height of the fall and how the chest strikes the ground (6). Yagmur et al. reported that falling from a height greater than 8 meters can cause substantial damage to the thoracic organs, with coronary artery dissection being a severe complication (7). Warner et al. showed that different landing positions were also crucial in influencing the injury and the part of the injury, with foot or hip landing accounting for 10%–40% of the landings that caused cardiac damage (8). Degeneration of intima and intra-arterial hypertension are the two key factors necessary for the development of arterial dissection. In this case, the patient was previously healthy and with no known risk factors for spontaneous coronary artery dissection. Given that the OCT suggested no structural coronary artery lesions, the likelihood of SCAD resulting from the patient's underlying disease or emotional stress was ruled out. Based on the patient's medical history, it was determined that the fall was most likely torso-first on the left side. The impact resulted in a significant increase in intrathoracic pressure due to the blunt force. It is well-established that the left anterior descending branch at the forefront of the heart is in the most vulnerable position. The rapid deceleration causes increased shear forces on the endothelium of the anterior descending coronary artery. In addition, it causes an acute increase in coronary blood pressure due to increased catecholamine levels in the body secondary to intense emotional stress. The combination of these two factors led to intimal tearing, resulting in coronary artery dissection (9, 10). Thus, there is a direct causal relationship between coronary artery dissection and trauma in this case.

The present case also demonstrates some diagnostic challenges associated with traumatic coronary dissections, especially in the setting of substantial chest trauma or multiple injuries. Due to mental status changes or sedation, the patient might not have been able to communicate symptoms of myocardial ischemia. Initially, the healthcare team considered rib fractures and violent chest impact as the cause of the pain. Repeated administration of Ibuprofen-Codeine Sustained Tablet might have masked coronary artery dissection following trauma to the anterior cardiac region. Unfortunately, the physicians did not identify this severe myocardial infarction in time. The Eastern Association for the Surgery of Trauma (EAST) guidelines recommend that healthcare providers have a high degree of suspicion for potential cardiac injury in patients with recent chest trauma who report chest pain or dyspnea or have evidence of significant thoracic trauma. Appropriate diagnostic testing includes a 12-lead electrocardiogram (ECG), the determination of troponin levels, and continuous ECG monitoring for a certain period (11). Our patient experienced recurring chest pain and a significant increase in cardiac enzymes, indicating the dynamic changes in cardiac enzymes after myocardial infarction. The presence of Q-wave formation, ST-segment elevation, and ST-segment depression in the mirror leads on the ECG raised a high suspicion of possible myocardial infarction and warranted urgent coronary angiography. When a filling defect on imaging is severe and persistent, it is classified as NHLBI staging type E, which poses a higher risk of coronary artery occlusion. For coronary arteries with persistent myocardial ischemia symptoms or electrocardiogram changes, and vessel diameter ≥2.5 mm, Percutaneous Coronary Intervention (PCI) is the first choice for patients with arterial dissection, which is an important measure to prevent acute vascular occlusion and ischemic complications. Antiplatelet therapy should be strengthened after operation (12).

Although coronary angiography is the primary method for the diagnosis of dissection, it can only observe two-dimensional images of the coronary artery in the longitudinal direction and is not effective in monitoring the floating endothelial pieces of the rupture, which is a significant limitation for determining the cause of entrapment and understanding the degree, nature, and extent of dissection (13). However, OCT allows for detailed visualization of ultrastructures such as intraluminal thrombus, pseudoterminal, and intramural hematomas to better assess vascular lesions and guide interventional treatment options (14). We employed OCT imaging to visualize the precise location and size of the arterial dissection, the length and area of the interstitial layer, and the area of the true lumen before the operation. This information helped us select the appropriate stent type and ensure proper stent apposition against the arterial wall. We also used OCT imaging postoperatively to assess the closure of the interstitial breach and the morphology of the residual hematoma to prevent adverse events such as re-accumulation of the hematoma and distant in-stent restenosis.

It is well-established that antiplatelet therapy should be prescribed after stenting to avoid acute thrombosis. Adequate antiplatelet therapy may increase bleeding risk after fracture dissection and internal fixation. If fracture surgery is performed before the diagnosis of coronary artery dissection, the patient may already be symptomatic due to a 90% compression of the anterior descending branch of the coronary artery. It is now understood that pain stimulates sympathetic excitation and leads to increased coronary artery wall tension, which may exacerbate coronary artery dissection and the risk of arrhythmia or sudden death due to the occlusion of the anterior descending branch. Therefore, the order in which fracture surgery and interventional surgery are carried out and the antithrombotic approach used during the transition between the two procedures can significantly affect patient prognosis. After thorough discussions between the orthopaedic and cardiovascular departments, it was determined that stent implantation should be prioritized, followed by administering indobufen and rivaroxaban as an antithrombotic measure post-surgery. To reduce the risk of bleeding, the medication would be discontinued 24 h before the fracture procedure and replaced with tirofiban, which has a shorter half-life. Following the surgery, the patient was prescribed aspirin and rivaroxaban to prevent thrombosis. Rivaroxaban was discontinued after six months.

## Conclusion

Our article outlines a case of coronary artery dissection resulting from trauma and comprehensively discusses the risk factors, pathophysiological mechanisms, treatment options, and mechanics involved. While treating patients with chest contusions, physicians should bear in mind that cardiac injury can occur, albeit rarely, and can be easily missed due to chest wall trauma. To prevent delays and ensure positive patient outcomes, physicians should regularly review the patient's electrocardiogram, cardiac ultrasounds, and cardiac enzymes during hospitalization. Furthermore, there is a need for additional research to understand how chest trauma leads to coronary artery dissection. If a patient has both a fracture and traumatic coronary artery dissection, physicians should carefully assess their vascular condition, bleeding risk, prognosis, and potential outcomes. The treatment plan and approach presented in this case may provide a clinical reference for similar situations.

## Data Availability

The original contributions presented in the study are included in the article/supplementary material, further inquiries can be directed to the corresponding authors.
